# Metabolic network rewiring of propionate flux compensates vitamin B12 deficiency in *C. elegans*

**DOI:** 10.7554/eLife.17670

**Published:** 2016-07-06

**Authors:** Emma Watson, Viridiana Olin-Sandoval, Michael J Hoy, Chi-Hua Li, Timo Louisse, Victoria Yao, Akihiro Mori, Amy D Holdorf, Olga G Troyanskaya, Markus Ralser, Albertha JM Walhout

**Affiliations:** 1 Program in Systems Biology, University of Massachusetts Medical School, Worcester, United States; 2 Program in Molecular Medicine, University of Massachusetts Medical School, Worcester, United States; 3 Department of Biochemistry, University of Cambridge, Cambridge, United Kingdom; 4 Department of Computer Science, Princeton University, Princeton, United States; 5 Lewis-Sigler Institute for Integrative Genomics, Princeton University, Princeton, United States; 6 Simons Center for Data Analysis, Simons Foundation, New York, United States; 7 The Francis Crick Institute, Mill Hill Laboratory, London, United Kingdom; Max Planck Institute of Molecular Cell Biology and Genetics, Germany

**Keywords:** metabolism, vitamin B12, propionate, network rewiring, transcription, bacteria, *C. elegans*, Human

## Abstract

Metabolic network rewiring is the rerouting of metabolism through the use of alternate enzymes to adjust pathway flux and accomplish specific anabolic or catabolic objectives. Here, we report the first characterization of two parallel pathways for the breakdown of the short chain fatty acid propionate in *Caenorhabditis elegans*. Using genetic interaction mapping, gene co-expression analysis, pathway intermediate quantification and carbon tracing, we uncover a vitamin B12-independent propionate breakdown shunt that is transcriptionally activated on vitamin B12 deficient diets, or under genetic conditions mimicking the human diseases propionic- and methylmalonic acidemia, in which the canonical B12-dependent propionate breakdown pathway is blocked. Our study presents the first example of transcriptional vitamin-directed metabolic network rewiring to promote survival under vitamin deficiency. The ability to reroute propionate breakdown according to B12 availability may provide *C. elegans* with metabolic plasticity and thus a selective advantage on different diets in the wild.

**DOI:** http://dx.doi.org/10.7554/eLife.17670.001

## eLife digest

Inborn errors of metabolism are human genetic diseases that cause developmental delays and are usually fatal. Propionic acidemia is an inborn error of metabolism where propionate, a byproduct created during the breakdown of fat and proteins, cannot be broken down efficiently. As a result, propionate builds up to toxic levels inside cells. Most animals, including humans, use a particular enzyme pathway to get rid of propionate. This pathway needs vitamin B12 in order to work, which is obtained from food.

Newborns are screened for propionic acidemia using a test that measures the levels of a molecule called 3-hydroxypropionate (3-HP) in the body. This molecule is not normally found in appreciable levels in healthy humans. However, it is not clear how 3-HP forms in individuals with propionic acidemia.

In 2014, researchers showed that in worms called *Caenorhabditis elegans*, propionate activates many genes when vitamin B12 levels are low. This suggests that the worms may have an alternate way to break down propionate when vitamin B12 is in short supply.

Now, Watson et al. – including some of the researchers involved in the 2014 work – have used a combination of genetic, computational and biochemical techniques to identify five genes that the worms use to break down propionate when vitamin B12 is not available. Furthermore, the level of 3-HP rises in worms that cannot use B12, just as is seen in humans with propionic acidemia. Thus, it appears that producing 3-HP may be an important step in an alternate pathway that does not require vitamin B12 to eliminate propionate.

Having an alternate way of breaking down propionate may be essential for *C. elegans* worms living in the wild, which have to adapt to changing dietary conditions that may or may not provide them with vitamin B12. Further studies are now needed to describe the metabolic effects of genes turned on by propionate and repressed by vitamin B12, and to investigate how propionate alters the activity of these genes.

**DOI:** http://dx.doi.org/10.7554/eLife.17670.002

## Introduction

Metabolic network rewiring to adjust metabolic flux in response to dietary or cellular cues can occur by transcriptional, post-transcriptional, or allosteric mechanisms (Desvergne et al., 2006). For instance, genes encoding enzymes involved in the breakdown of galactose in the Leloir pathway are activated in yeast and other organisms upon a shift from glucose to galactose as a carbon source (Fridovich-Keil, 2006). As a second example, in both yeast and humans, glycolytic flux is temporarily re-routed through the pentose phosphate pathway to provide a first-line protection against oxidative stress (Stincone et al., 2014). However, metabolic network rewiring to compensate for the absence of a vitamin or due to the toxic accumulation of a cellular metabolite has not yet been described.

**Figure 1.**
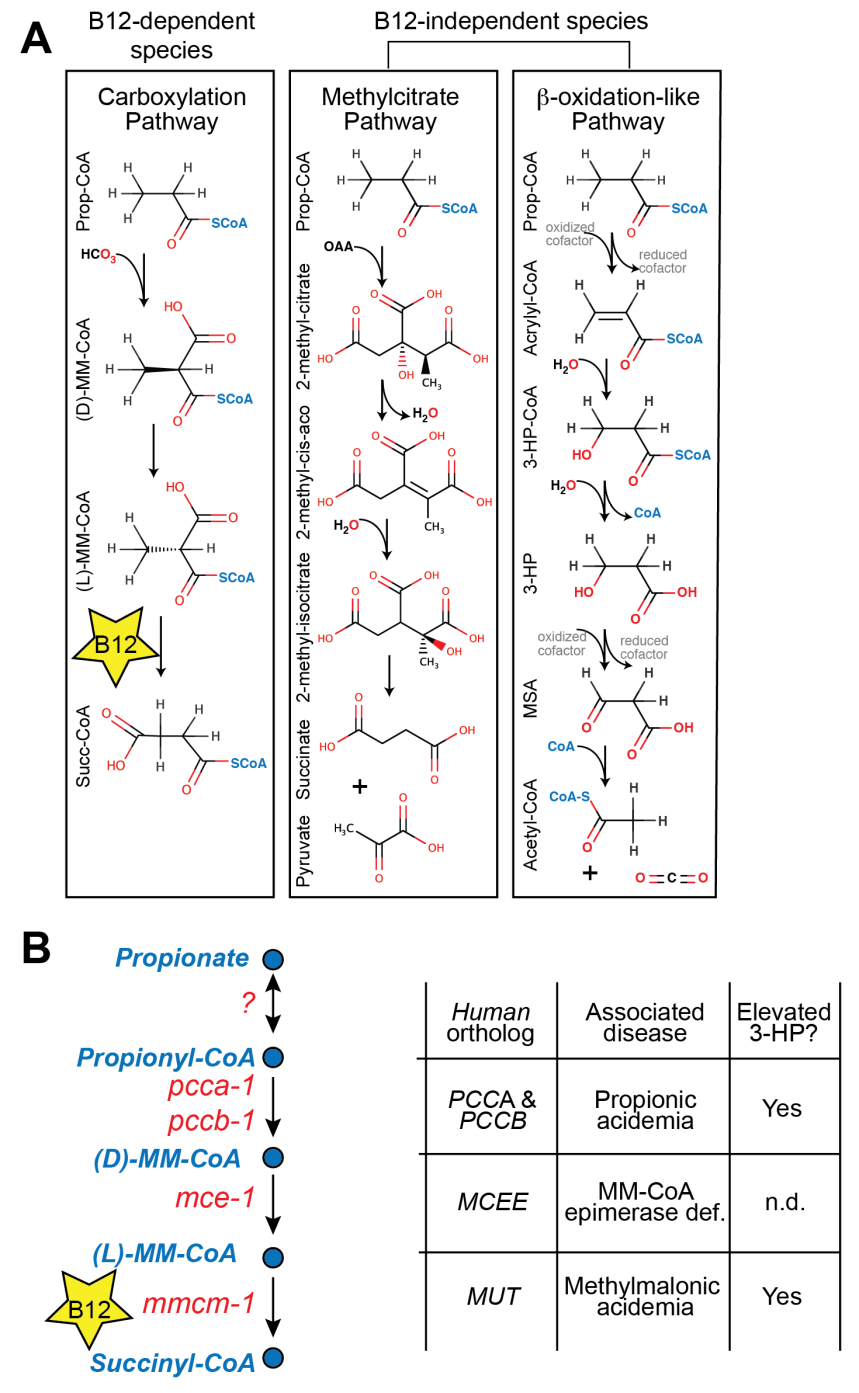
Propionate breakdown pathways in different organisms. (**A**) Vitamin B12-dependent species use a propionate carboxylation pathway to breakdown propionate. Other species use either the methylcitrate pathway or a β-oxidation-like pathway. (**B**) Diagram of canonical vitamin B12-dependent propionyl-CoA breakdown pathway indicating *C. elegans* and human enzymes and associated genetic diseases. MM – methylmalonyl, 3-HP – 3-hydroxypropionate, MSA – malonic semialdehyde, n.d. – not determined. **DOI:** http://dx.doi.org/10.7554/eLife.17670.003

In both mammals and the nematode *C. elegans*, vitamin B12 is a critical cofactor in the canonical propionyl-CoA breakdown pathway (Figure 1A and 1B). Propionyl-CoA is produced during the catabolism of odd chain fatty acids and branched chain amino acids, and is interconverted with the short chain fatty acid propionate derived from bacterial fermentation of dietary fibers in the intestine (Kasubuchi et al., 2015). Many organisms, however, do not utilize vitamin B12 in the breakdown of propionate. For instance, *Saccharomyces cerevisiae* utilizes the methylcitrate cycle, whereas plants and *Candida albicans* use a β-oxidation-like pathway (Halarnkar and Blomquist, 1989; Otzen et al., 2014) (diagrammed in Figure 1A).

Mutations in genes in the canonical vitamin B12-dependent propionate breakdown pathway cause propionic- and methylmalonic acidemias, diseases in which propionate and its derivatives accumulate to toxic levels (La Marca et al., 2007). These diseases are diagnosed by elevated levels of specific metabolites such as 3-hydroxypropionate (3-HP), which is not normally detected at appreciable levels in healthy individuals (Matsumoto and Kuhara, 1996) (Figure 1B). Interestingly, 3-HP is an intermediate in the β-oxidation-like propionate breakdown pathway found in some vitamin B12-independent organisms (Figure 1A). This observation suggests that propionic- and methylmalonic acidemia patients may break down propionate to some extent via an alternate oxidative route (Ando et al., 1972).

We previously identified numerous *C. elegans* metabolic genes that are transcriptionally repressed in response to vitamin B12 (MacNeil et al., 2013; Watson et al., 2014). This finding suggests that the *C. elegans* metabolic network is differentially wired under vitamin B12-deficient versus vitamin B12-replete nutritional conditions. However, the biological significance of the transcriptional rewiring by the vitamin B12/propionate axis remains unknown.

Here, we find that *C. elegans* transcriptionally activates a β-oxidation-like propionate breakdown shunt under vitamin B12-deficient dietary conditions, or under genetic conditions mimicking propionic- or methylmalonic acidemia. This pathway is chemically similar to, but genetically distinct from the pathway found in *Candida albicans*. We detect elevated 3-HP in animals with a dysfunctional canonical propionate breakdown pathway, demonstrating that the *C. elegans* model faithfully recapitulates a metabolic phenotype of propionic- and methylmalonic acidemia.

*C. elegans* is likely to encounter both vitamin B12-replete and B12-deficient diets in the wild because only a minority of bacterial species synthesize vitamin B12 (Karasseva et al., 1977; Sañudo-Wilhelmy et al., 2014). We find that activation of the *C. elegans* propionate shunt enables survival on vitamin B12-deficient diets. Altogether, our data suggest that metabolic network rewiring in response to vitamin B12 status enables the animal to thrive both when dietary vitamin B12 is low, and when this cofactor is in ample supply. This metabolic plasticity likely confers a selective advantage and evolutionary benefit.

## Results

### A *C. elegans* model of propionic acidemia

Patients with propionic acidemia harbor loss of function mutations in both alleles of either PCCA or PCCB, which encode the two members of the propionyl-CoA carboxylase complex that catalyzes the first reaction in the canonical propionate breakdown pathway (Deodato et al., 2006) (Figure 1A and B). These patients suffer from the toxic effects of propionate buildup, which manifest in several organ systems and lead to acute symptoms such as poor feeding, vomiting, hypotonia, lethargy, seizures, failure to thrive, intellectual disability, pancreatitis and cardiomyopathy (Carrillo-Carrasco and Venditti, 2012).

**Figure 2.**
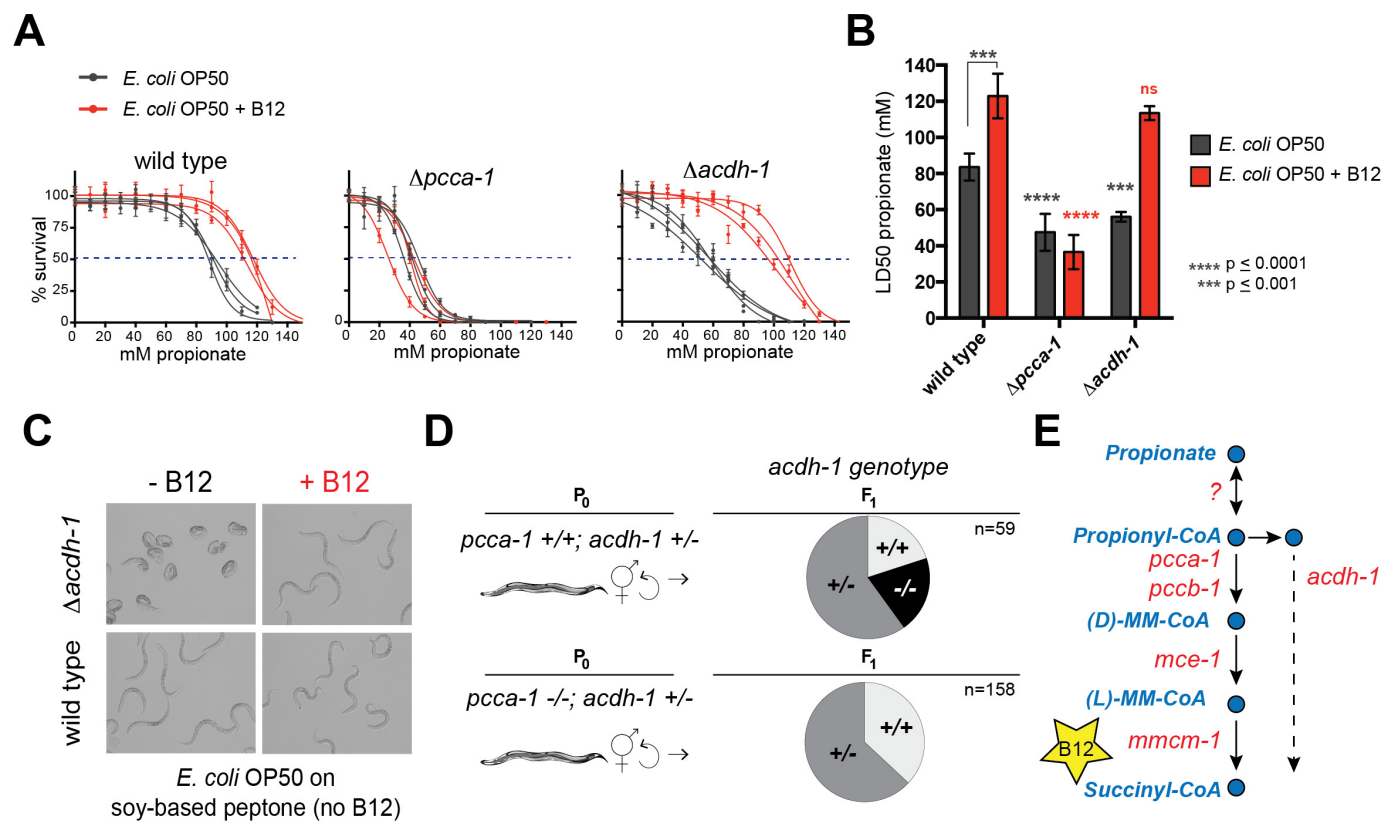
*acdh-1* mutants are sensitive to propionate and synthetic lethal with *pcca-1* mutants. (**A**) Dose-response curves showing that Δ*pcca-1* and Δ*acdh-1* mutants exhibit increased sensitivity to propionate compared to wild type animals. Three biological replicate experiments are shown, each with three technical replicates per data point with average and SEM plotted. (**B**) Average LD_50_ and standard deviation of data shown in (**A**). Unpaired student’s T tests were used to calculate p-values. Black asterisks indicate significant difference compared to wild type, red asterisks indicate significant difference compared to wild type plus B12. (**C**) Δ*acdh-1* mutants cannot survive on *E. coli* grown in vitamin B12 deficient media. (**D**) Δ*pcca-1* and Δ*acdh-1* are synthetically lethal because a cross between Δ*pcca-1* and Δ*acdh-1* mutants yielded no viable double homozygous mutants. *pcca-1* +/+;*acdh-1* +/- animals and *pcca-1 -/-;acdh-1* +/- animals were grown on *E. coli* OP50 seeded plates containing 64nM vitamin B12, and individual F1s were picked onto new plates, also containing 64nM vitamin B12. The distribution of *acdh-1* genotypes among the viable F1s picked from each P0 genotype is shown. (**E**) These genetic data support a role for *acdh-1* parallel to the canonical propionate breakdown pathway. **DOI:** http://dx.doi.org/10.7554/eLife.17670.004

Deletion of the *C. elegans* ortholog of PCCA, *pcca-1*, slows development rate (Watson et al., 2014) and renders animal sensitive to propionate-induced toxicity: the LD_50_ of wild type animals is ~80mM propionate, while the LD_50_ of Δ*pcca-1* mutants is ~45 mM (Figure 2A and B). As expected, vitamin B12 supplementation to wild type animals increases propionate tolerance on the low-B12 *E. coli* OP50 diet (Watson et al., 2014), whereas it has no beneficial effect in Δ*pcca-1* animals (Figure 2A and B).

### *pcca-1* is synthetic lethal with *acdh-1*

The *C. elegans* acyl-CoA dehydrogenase *acdh-1* is differentially expressed depending on the vitamin B12/propionate axis: its transcript levels are very low when vitamin B12 is high, and increase several hundred fold in response to propionate accumulation (Watson et al., 2013; 2014). A null mutation in *acdh-1* also renders *C. elegans* sensitive to propionate: the LD_50_ in these animals is ~50 mM (Figure 2A and B). However, in contrast to Δ*pcca-1* mutants, propionate sensitivity in Δ*acdh-1* mutants is completely rescued by vitamin B12 supplementation (Figure 2A and B). Furthermore, Δ*acdh-1* mutants exhibit embryonic lethality on a very low-vitamin B12 diet (*E. coli* OP50 grown on soy-peptone), and this phenotype can also be rescued by supplementing vitamin B12 (Figure 2C). Acyl-CoA dehydrogenases catalyze the first step in β-oxidation of fatty acids (Berg et al., 2012). Therefore, we hypothesized that *acdh-1* may function in an alternate β-oxidation-like propionate breakdown pathway, hereafter referred to as the 'propionate shunt', to enable survival of the animal on vitamin B12-deficient diets.

Genes in parallel pathways often exhibit synthetic phenotypes (Clark et al., 1994; Costanzo et al., 2010). If *acdh-1* does function in a propionate shunt, one would expect the propionate sensitivity to further increase when both *acdh-1* and the canonical propionate-breakdown pathway are perturbed. To test this, we attempted to generate double null mutants that harbor deletions in both *acdh-1* and in *pcca-1*. However, a cross between Δ*pcca-1* and Δ*acdh-1* mutants yielded no viable double homozygous mutant offspring (Figure 2D and Supplementary file 1). This finding demonstrates that loss of function in both *pcca-1* and *acdh-1* results in synthetic lethality, and supports the hypothesis that *acdh-1* functions in a parallel propionate breakdown pathway (Figure 2E).

### A synthetic lethality screen identifies additional propionate shunt genes

**Figure 3.**
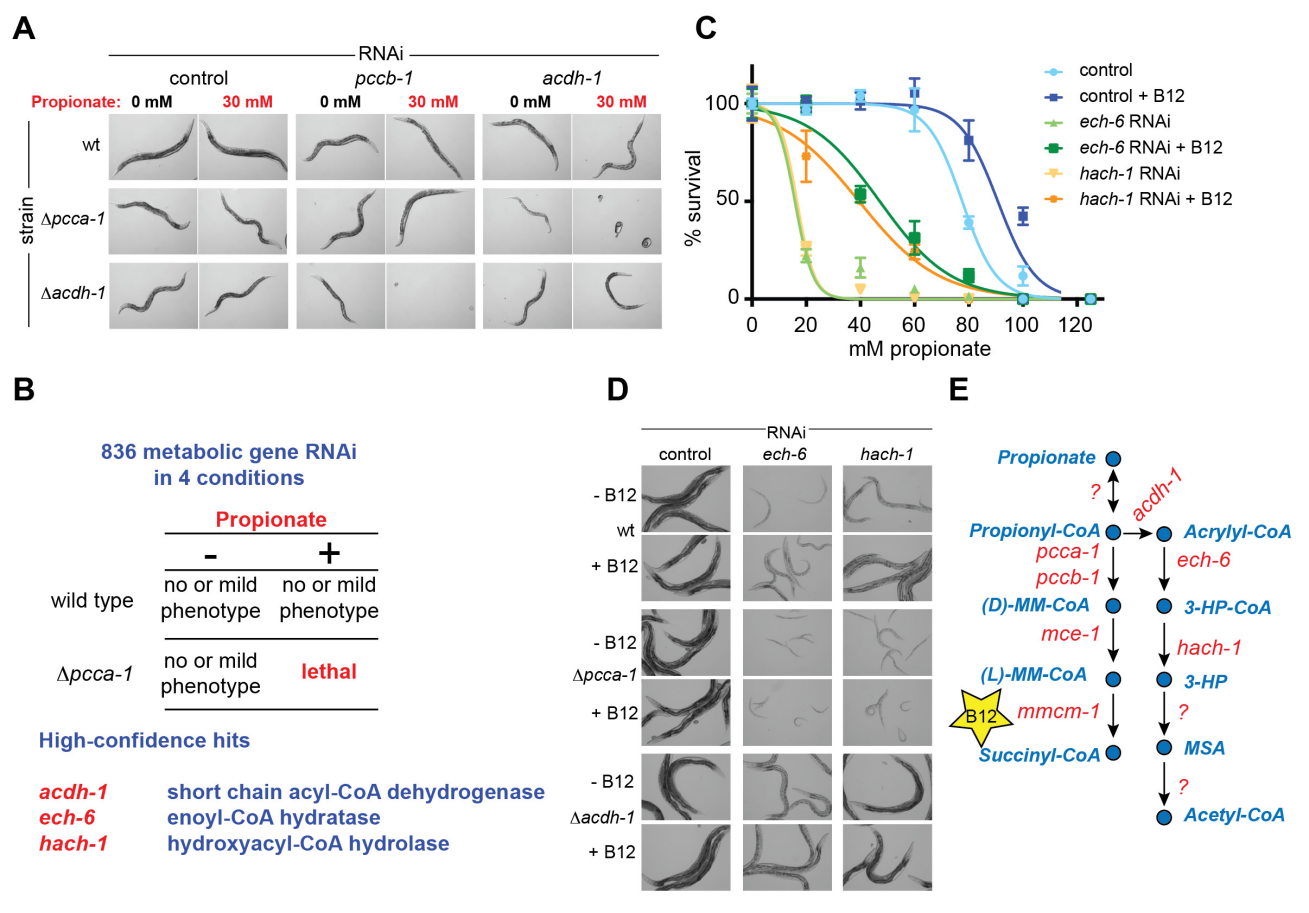
A synthetic genetic interaction screen identifies candidate genes involved in the propionate shunt. (**A**) RNAi of *acdh-1* is lethal in Δ*pcca-1* mutants supplemented with 30 mM propionate. (**B**) Synthetic genetic interaction screen of 836 metabolic genes in presence or absence of 30 mM added propionate, in wild type and Δ*pcca-1* mutant animals identifies three candidate genes, including *acdh-1*. (**C**) Propionate toxicity dose response curve showing that the two candidate genes identified in the screen, *ech-6* and *hach-1*, phenocopy *acdh-1* loss-of-function. (**D**) Genetic buffering of *ech-6* and *hach-1* RNAi phenotypes by loss of *acdh-1*. Representative images of animals subjected to two generations of RNAi knockdown are shown. (**E**) Our data indicate that *ech-6* and *hach-1* function downstream of *acdh-1* in the propionate breakdown shunt. **DOI:** http://dx.doi.org/10.7554/eLife.17670.005

To identify additional *C. elegans* genes that may function in a pathway with *acdh-1*, we performed a synthetic genetic interaction screen using Δ*pcca-1* mutants and an RNAi library of 836 *C. elegans* metabolic genes (Supplementary file 2). RNAi of *acdh-1* in the Δ*pcca-1* mutant resulted in complete lethality in the presence of 30 mM propionate (Figure 3A). Therefore, we screened for knockdowns that led to non-viable offspring in the Δ*pcca-1* mutant supplemented with 30 mM propionate. Only three high-confidence hits were obtained from this screen: *acdh-1* itself*, ech-6*, an enoyl-CoA hydratase 6, and F09F7.4, which we named *hach-1*, for hydroxyacyl-CoA hydrolase (Figure 3B). Enoyl-CoA hydratases function in the second step of β-oxidation (Berg et al., 2012) and, therefore, *ech-6* is an excellent candidate to catalyze the second reaction in the propionate shunt, directly downstream of *acdh-1*. In the vitamin B12-independent yeast *C. albicans*, the Ehd3 enzyme converts 3-hydroxypropionyl-CoA into 3-HP and CoA in the third step of the β-oxidation-like propionate breakdown pathway (Otzen et al., 2014). Ehd3 is the one-to-one ortholog of *hach-1*, the third gene we identified, which we therefore placed downstream of *ech-6*. Importantly, knockdown of either *ech-6* or *hach-1* resulted in similar phenotypes compared to loss of *acdh-1*: increased propionate sensitivity that was partially rescued by the addition of vitamin B12 (Figure 3C). This observed phenocopying, along with the co-synthetic lethality with *pcca-1*, supports the hypothesis that *acdh-1, ech-6* and *hach-1* function together in a genetic pathway.

The first reaction of the propionate shunt produces acrylyl-CoA, a highly toxic and reactive metabolite (Hellwig et al., 1993; Saillenfait et al., 1999). Since we predict that acrylyl-CoA is the substrate of ECH-6, we hypothesized that *ech-6* perturbation would result in a severe phenotype due to toxic buildup of acrylyl-CoA or its hydrolyzed derivative acrylate. Indeed, RNAi of *ech-6*, and to a lesser extent *hach-1,* strongly reduces *C. elegans* growth and viability (Figure 3D). This phenotype was partially rescued by vitamin B12 supplementation, which facilitates propionate flux through the canonical pathway (Figure 3D). This rescue depends on a functional canonical B12-dependent propionate breakdown pathway, as vitamin B12 supplementation had no beneficial effect when *ech-6* or *hach-1* was knocked down in Δ*pcca-1* mutants (Figure 3D).

We found that loss of *acdh-1* largely suppressed the phenotypic effects of *ech-6* and *hach-1* knockdown, likely due to the lack of acrylyl-CoA production in the absence of *acdh-1* (Figure 3D). This genetic buffering supports the placement of *ech-6* and *hach-1* downstream of *acdh-1* in the propionate shunt (Figure 3E).

### *hphd-1* and *alh-8* function in the propionate shunt

**Figure 4.**
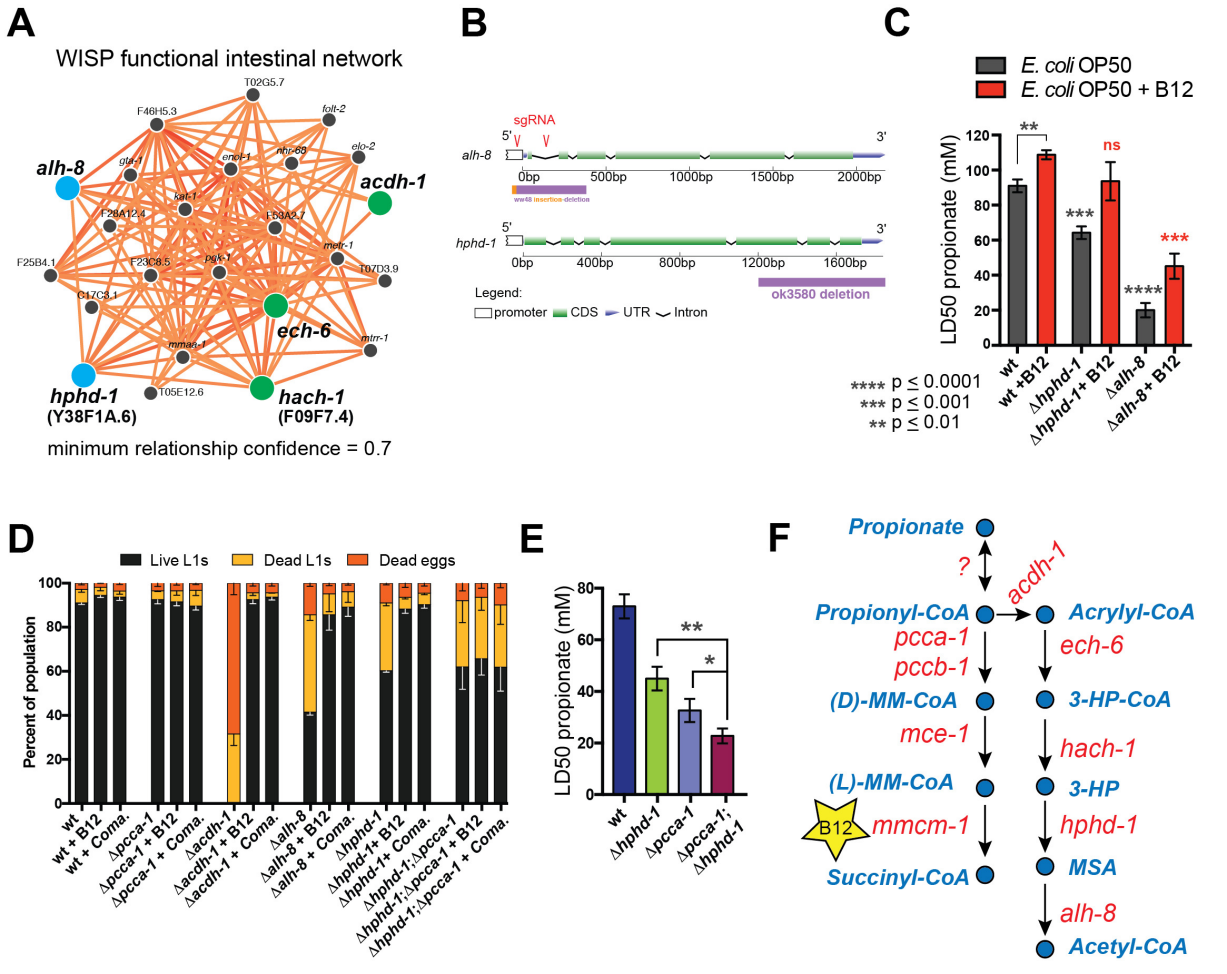
Identifying additional putative propionate shunt genes. (**A**) *hphd-1* and *alh-8* (blue) are tightly connected to *acdh-1, ech-6* and *hach-1* (green) in a *C. elegans* intestinal functional network and are candidates to catalyze the fourth and fifth reactions of the propionate shunt, respectively. (**B**) Structure of CRISPR/Cas9-generated *alh-8* mutant. Diagram of the mutation generated by CRISPR/Cas9-mediated genome editing using an sgRNA (red sequence) targeting *alh-8*. The *alh-8*(ww48) mutation consists of a 23 bp insertion and 399 bp deletion, and removes a part of the 5’UTR, the start codon, the first and second exons, and part of the third exon. Also shown is the Δhphd-1(ok3590) mutation. (**C**) Propionate toxicity dose response showing that Δ*hphd-1* and Δ*alh-8* mutants phenocopy *acdh-1, ech-6* and *hach-1* perturbation. (**D**) Δ*hphd-1* and Δ*alh-8* mutants exhibit partial lethality on low-B12 conditions. Like the Δ*acdh-1* mutant phenotype, Δ*hphd-1* and Δ*alh-8* mutant phenotypes were rescued by 64nM B12 supplementation or by *Comamonas aquatica* DA1877 (*Coma.*). The partial lethal phenotype of the Δ*hphd-1*;Δ*pcca-1* double mutant was not rescued by B12. (**E**) Combined deletion of *hphd-1* and *pcca-1* renders the animals more sensitive to propionate than mutation in either gene alone. Note that Δ*hphd-1* may not be a null allele. (**F**) The *C. elegans* propionate breakdown shunt pathway comprises five genes: *acdh-1, ech-6, hach-1, hphd-1* and *alh-8*. **DOI:** http://dx.doi.org/10.7554/eLife.17670.006

The β-oxidation-like propionate breakdown pathway includes two additional reactions that convert the third metabolic intermediate 3-hydroxypropionate (3-HP) to malonic semialdehyde (MSA) and finally to acetyl-CoA and CO_2_ (Figure 1A). Importantly, the gene encoding the enzyme that converts 3-HP into MSA has not yet been identified in any metazoan. To identify enzymes that may catalyze the last two reactions in the *C. elegans* propionate shunt, we utilized WISP, a server for predicting tissue-specific functional networks based on the integration of a large compendium of diverse datasets (http://wisp.princeton.edu, Yao et al., in preparation; V. Yao, personal communication, June 2016). The top predicted functional connections to *acdh-1, ech-6* and *hach-1* included the metabolic genes Y38F1A.6 and *alh-8* (Figure 4A), neither of which was tested in genetic interaction screen because they were not included in the ORFeome RNAi library (Supplementary file 2). These genes encode excellent candidate enzymes to catalyze the fourth and fifth reactions of the propionate shunt, respectively. Y38F1A.6 is the ortholog of human ADHFE1 (also known as HOT), a hydroxyacid-oxoacid transhydrogenase that has been found to metabolize β-hydroxybutyrate (GHB), a structural analog of 3-HP (Lyon et al., 2009). We will henceforth refer to Y38F1A.6 as *hphd-1 *(3-hydroxypropionate-oxoacid transhydrogenase). ALH-8 is homologous to human ALDH6A1, a decarboxylating dehydrogenase predicted to act on two structurally similar metabolites: methylmalonic semialdehyde from valine breakdown (Marcadier et al., 2013; Sass et al., 2012), and malonic semialdehyde (Marcadier et al., 2013), the substrate in the fifth reaction of the propionate oxidation pathway (Figure 1A). Additionally, *hphd-1* and *alh-8* are predicted to localize to the mitochondria along with *acdh-1, ech-6*, and *hach-1* (Yilmaz and Walhout, 2016; http://wormflux.umassmed.edu/).

**Figure 4—figure supplement 1.**
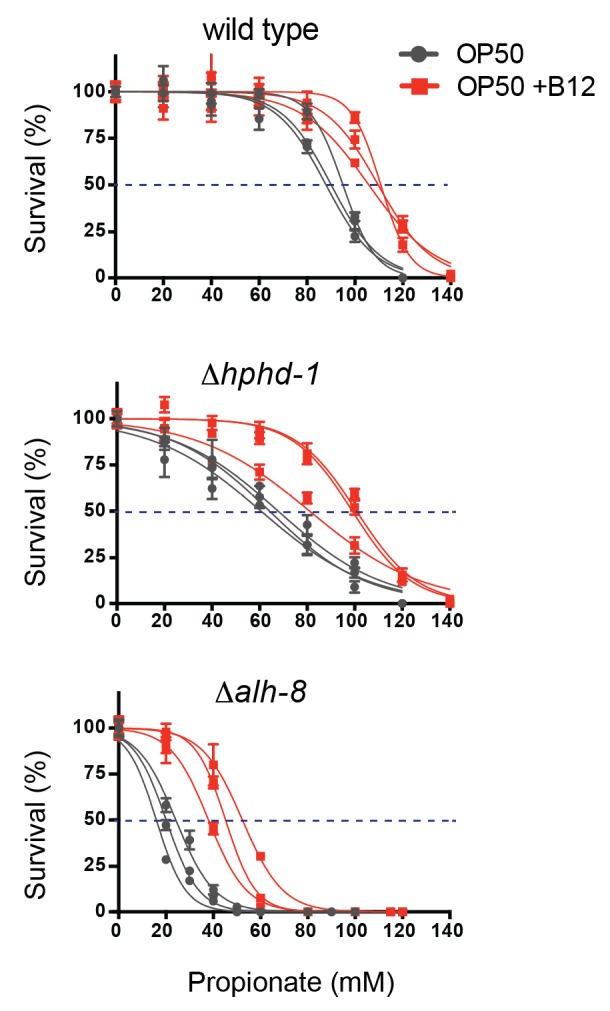
*Δhphd-1* and *Δalh-8* mutants exhibit increased sensitivity to propionate compared to wild type animals. Three biological replicate corves are shown. **DOI:** http://dx.doi.org/10.7554/eLife.17670.007

We obtained an *hphd-1* deletion mutant from the *C. elegans* genetics center, and generated an *alh-8* deletion mutant by CRISPR/Cas9-mediated genome editing (Kim et al., 2014) (Figure 4B). Both mutants phenocopied *acdh-1, ech-6* and *hach-1* loss of functions: they exhibited decreased propionate tolerance that was at least partially rescued by vitamin B12 supplementation (Figure 4C, Figure 4—figure supplement 1). Both Δ*hphd-1* and Δ*alh-8* mutants displayed partial lethality on low-B12 diets, which was rescued by vitamin B12 supplementation (Figure 4D). This rescue was dependent on a functional canonical propionate breakdown pathway, as vitamin B12 failed to rescue the partial larval lethality exhibited by the double Δ*hphd-1*;Δ*pcca-1* mutant (Figure 4D). This result indicates that activation of the propionate shunt is required to sustain viability. Δ*hphd-1*;Δ*pcca-1* mutants also exhibited increased propionate sensitivity compared to either single mutant, indicating a conditional genetic interaction between *hphd-1* and *pcca-1,* but not complete lethality like the Δ*acdh-1;*Δ*pcca-1* double mutant (Figure 4E). This may be due to Δ*hphd-1* not being completely null, or it is possible that an intact half-pathway is sufficient for at least partial survival. Altogether, these observations support the placement of *hphd-1* and *alh-8* in the same pathway as *acdh-1* (Figure 4F).

### Propionate shunt genes are repressed by vitamin B12 and activated by propionate

**Figure 5.**
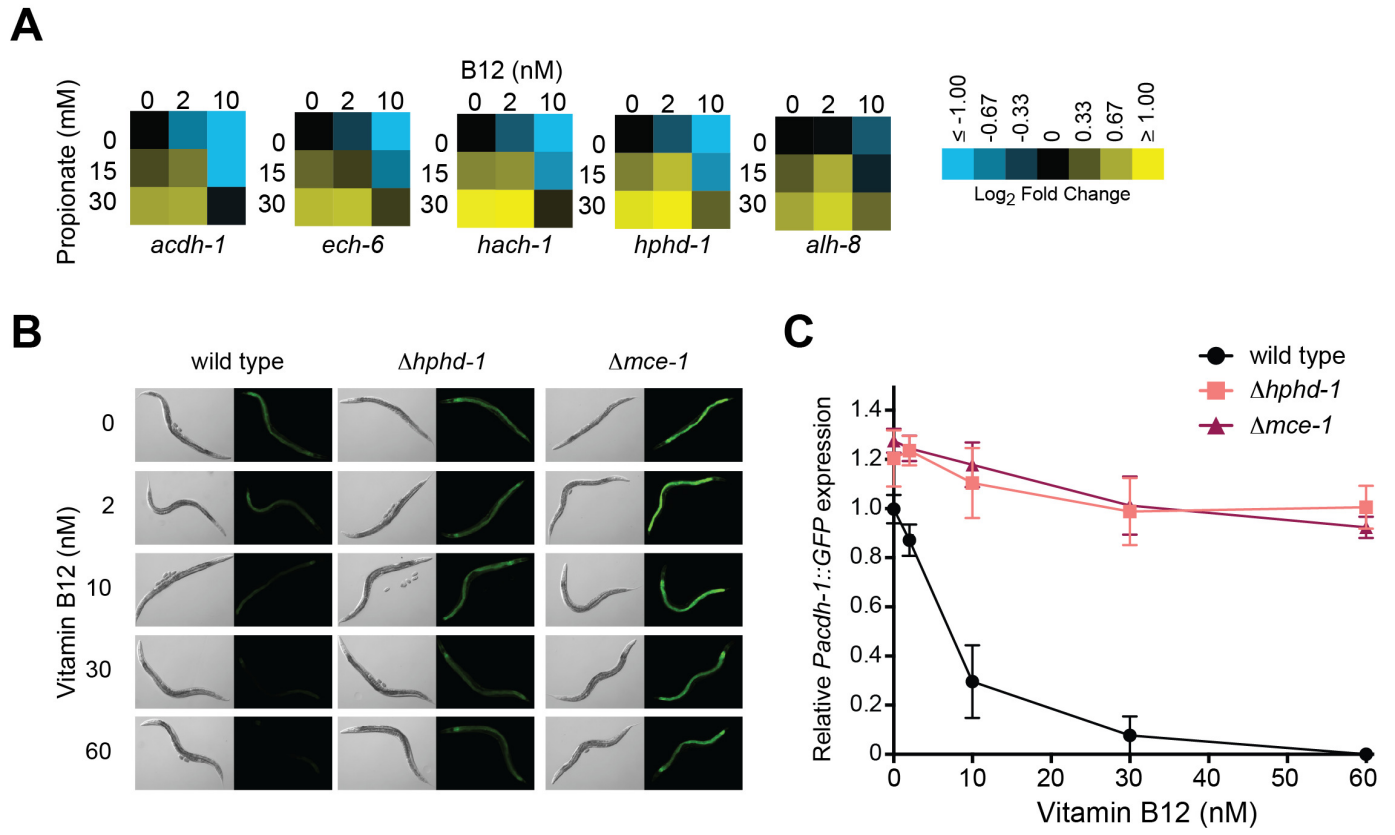
Transcriptional activation of the propionate shunt. (**A**) The expression of all five propionate shunt genes is repressed by vitamin B12 and activated by propionate. Condition matrices are shown for each shunt gene. Expression is normalized to levels in the control condition (no vitamin B12, no propionate). (**B**) Vitamin B12 reduces GFP levels in *Pacdh-1::GFP* transgenic animals, but not in those carrying a deletion in the canonical propionate pathway gene *mce-1* or in the propionate shunt gene *hphd-1*. (**C**) Quantification of GFP levels from part (**B**) **DOI:** http://dx.doi.org/10.7554/eLife.17670.008

We previously found reduced transcript levels of each of the five genes encoding propionate shunt enzymes in response to the vitamin B12-synthesizing bacteria *Comamonas aquatica* (MacNeil et al., 2013). However, under genetic conditions mimicking propionic acidemia (*i.e.,* when the animals cannot use vitamin B12 to breakdown propionate), vitamin B12 fails to reduce *acdh-1* expression (Watson et al., 2014). This observation led to the hypothesis that, rather than directly sensing vitamin B12 levels, the *C. elegans* gene regulatory network responds to elevated levels of propionate or propionyl-CoA (or a derivative thereof) to activate the shunt. Indeed, we found that reduced expression of all five shunt genes by vitamin B12 is reversed by supplementation of excess propionate (Figure 5A).

Activation of *acdh-1* in response to propionate buildup occurs through its 1.5 kb promoter, indicating that it is governed by transcriptional mechanisms (Watson et al., 2014). Not only is the *acdh-1* promoter activated by propionate and by canonical pathway perturbations, it is also activated by perturbation of the propionate shunt genes *ech-6, hach-1* and *acdh-1* itself (Watson et al., 2013). To determine whether a deletion in *hphd-1* also activates the *acdh-1* promoter, we crossed the Δ*hphd-1* mutant to a transgenic strain expressing the green fluorescent protein (GFP) under the control of the *acdh-1* promoter. Loss of *hphd-1* did in fact lead to greater *acdh-1* promoter activity, providing additional evidence that *hphd-1* functions in propionate breakdown (Figure 5B and C).

### 3-Hydroxypropionate is a substrate for HPHD-1

**Figure 6.**
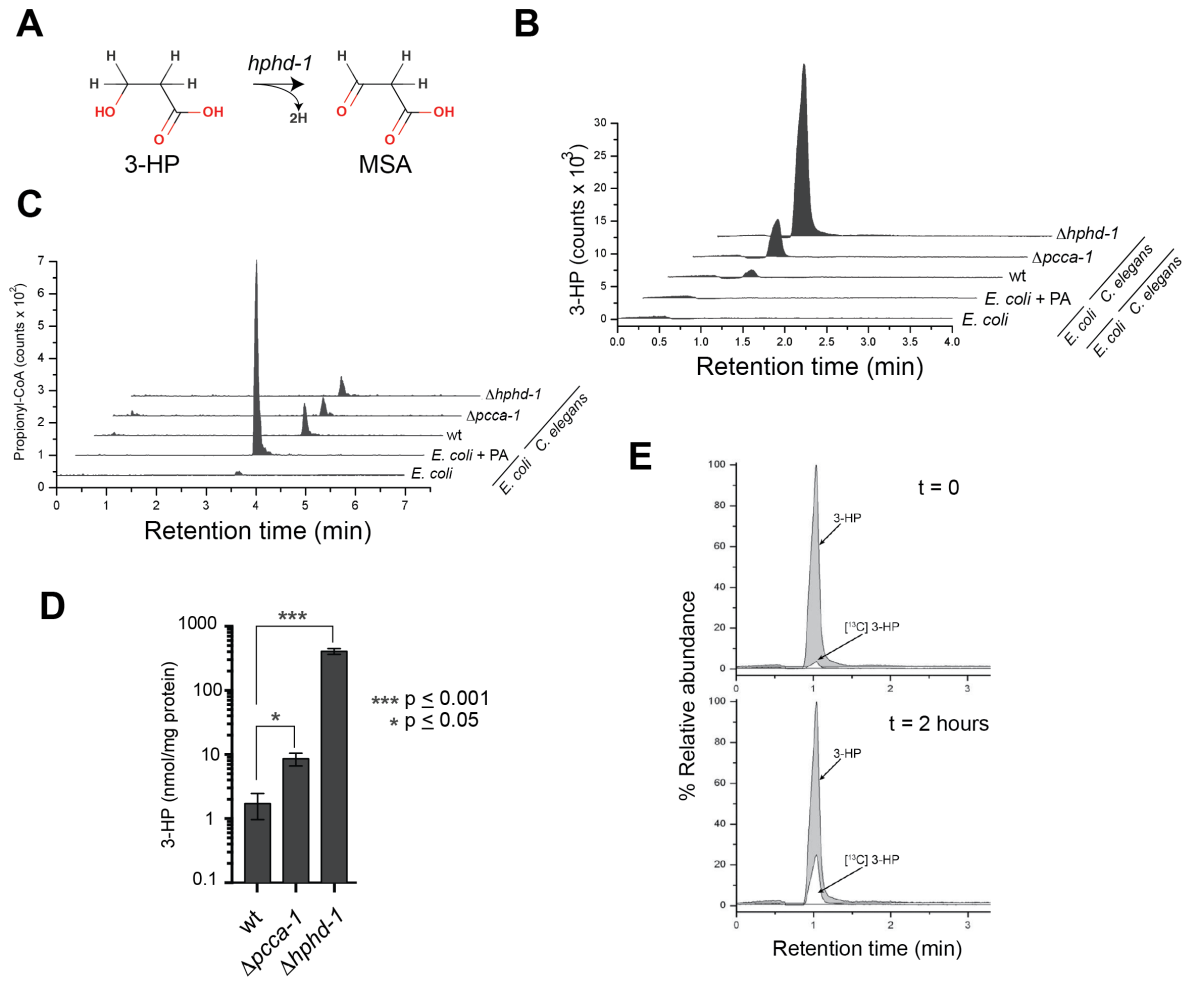
3-Hydroxypropionate is a substrate for HPHD-1. (**A**) Conversion of 3-hydroxypropionate (3-HP) into malonicsemialdehyde (MSA). (**B**) 3-HP mass spectrometry chromatogram for wild type, Δ*pcca-1* and Δ*hphd-1* animals. 3-HP was not detected in *E. coli* OP50 with or without supplemented propionate. (**C**) Propionyl-CoA chromatograms from *E. coli* and *C. elegans* samples. Propionyl-CoA quantifications are as follows: 1.86, 0.20, 0.14, and 0.13 nmol/mg protein for *E coli* + PA, wild type *C. elegans*, Δ*pcca-1* and Δ*hphd-1* mutants, respectively. For *E. coli* -PA, propionyl-CoA was detectable but not quantifiable in our assay. (**D**) Average 3-HP quantities normalized to total protein levels from three biological replicates, +/- SEM. Animals were grown on *E. coli* OP50. (**E**) ^13^C-labeled propionate fed to Δ*hphd-1* mutant animals for 2 hr yielded ^13^C-labeled 3-HP, demonstrating that *C. elegans* oxidizes propionate to 3-HP. Shown are SRM (MS^2^) chromatograms specific for 3-HP. The peak corresponding to the natural ^13^C isotope distribution (~ 1.1% of ^12^C signal) is illustrated for comparison in t = 0. **DOI:** http://dx.doi.org/10.7554/eLife.17670.009

3-HP is a unique metabolic intermediate produced by the propionate oxidation pathway: to our knowledge neither KEGG, nor any other metabolic database, lists this metabolite being produced by any other metabolic pathway in metazoans, though it can be produced through several different pathways in microorganisms. The fourth reaction in the propionate shunt involves the conversion of 3-HP into MSA (Figure 6A). Annotated with the enzyme commission number EC 1.1.1.59, the gene encoding this enzyme has, to our knowledge, not yet been identified in any metazoan. Our co-expression network analysis and subsequent genetic investigation identified HPHD-1 as a candidate for this enzyme. If true, we would predict that 3-HP accumulates in the Δ*hphd-1* mutant.

Using liquid chromatography/selective reaction monitoring mass spectrometry (LC-SRM) we detected 3-HP in *C. elegans,* but not in its *E. coli* diet (Figure 6B). We did detect ample propionyl-CoA in *E. coli* supplemented with propionate, so the lack of 3-HP in *E. coli* was not due to lack of pathway substrate (Figure 6C). Therefore, we conclude that the 3-HP detected is derived from *C. elegans* and not from its bacterial diet. We observed a >4-fold increase in 3-HP levels in Δ*pcca-1* mutants, which mirrors elevated 3-HP levels observed in human patients with propionic acidemia caused by PCCA or PCCB mutations (Carrillo-Carrasco and Venditti, 2012), and confirms that the propionate shunt is active when the canonical pathway is perturbed (Figure 6B and D). Importantly, we detected a >200-fold increase in 3-HP levels in Δ*hphd-1* mutants, which supports our prediction that HPHD-1 metabolizes 3-HP under low B12 conditions (Figure 6B and D). To verify that 3-HP is indeed derived from propionate, we performed carbon tracing by feeding Δ*hphd-1* mutant animals *E. coli* OP50 supplemented with ^13^C-propionate. We detected the formation of ^13^C-3-HP after 2 hr, demonstrating that *C. elegans* indeed converts propionate to 3-HP (Figure 6E).

### Human homologs of *C. elegans* shunt genes are activated by propionate

**Figure 7.**
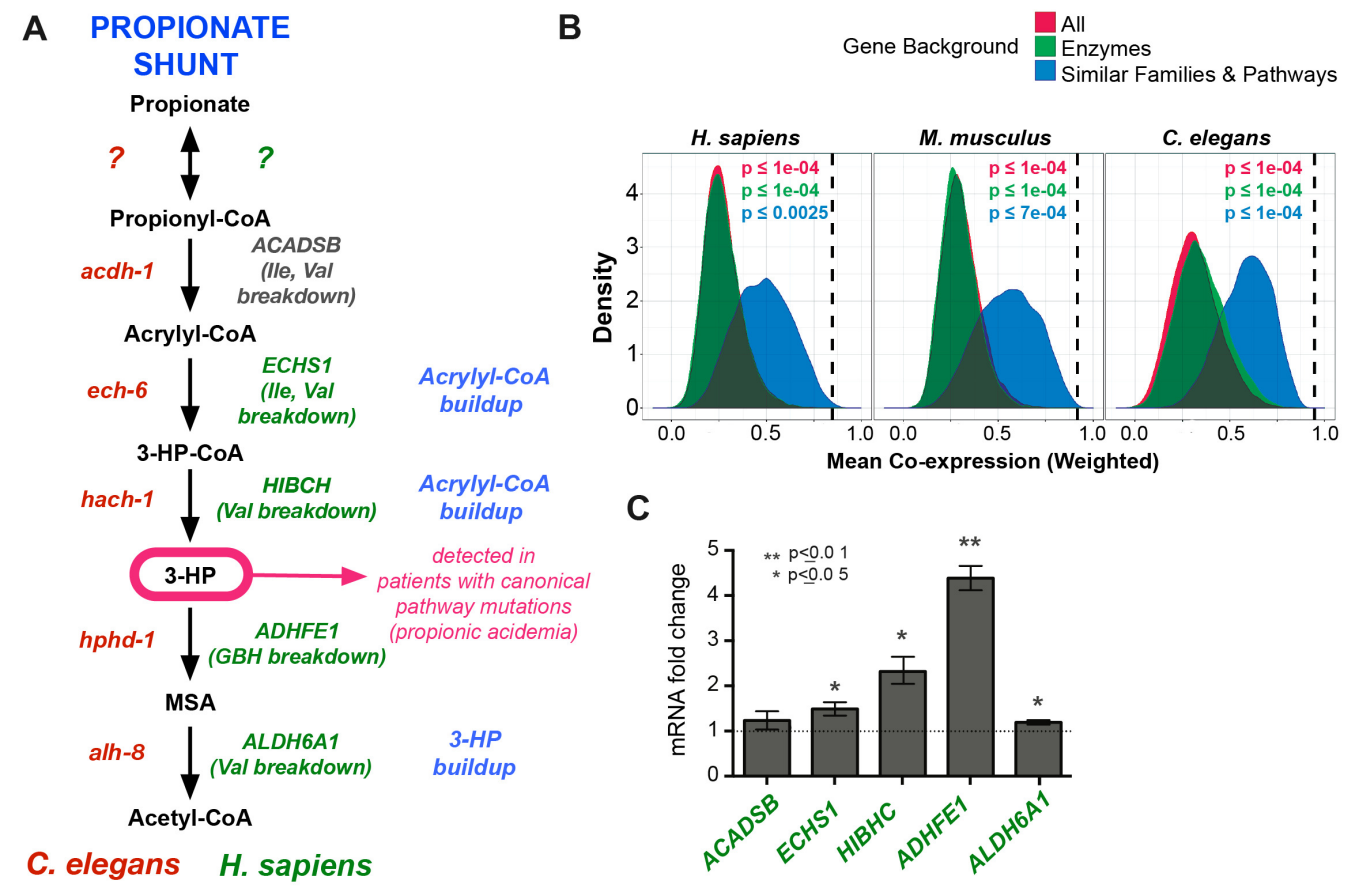
Comparison between putative human and *C. elegans* propionate shunts. (**A**) Comparison between *C. elegans* propionate shunt genes (red) and candidate human shunt genes (green, gray). Green text indicates higher confidence annotations based on patient mutations and metabolomics, or in the case of ADHFE1 one-to-one orthology of unique enzymes in both genomes. 3-HP is marked in magenta to indicate that it is a biomarker for impaired flux in the canonical, vitamin B12-dependent propionate breakdown pathway, such as occurs in patients with propionic or methylmalonic acidemia. (**B**) *C. elegans* propionate shunt genes and orthologs in mouse and humans are strongly co-expressed as a group compared to 10,000 random permutations of five genes from either the whole genome, a subset of only metabolic genes, or a subset of related metabolic genes from connected pathways, including BCAA breakdown and the TCA cycle. The expression data used for this analysis was compiled and weighted using the SEEK and modSEEK databases. Distributions of co-expression scores are shown for each set of randomizations, and vertical dashed lines indicate actual weighted co-expression score for propionate shunt genes and orthologs in human, mouse and *C. elegans*. (**C**) The expression of several candidate human genes is activated in response to propionate in HepG2 liver carcinoma cells. qRT-PCR experiment showing the average of four replicate experiments, each containing three technical replicates. **DOI:** http://dx.doi.org/10.7554/eLife.17670.010

3-HP is a specific diagnostic marker of propionic- and methylmalonic acidemias, as it is uniquely elevated in these diseases. This suggests that an alternative propionate breakdown pathway may be operational in humans as well, at least in patients with impaired canonical propionate breakdown. Interestingly, the closest human homologs of the *C. elegans* shunt enzymes are known to catalyze structurally similar reactions in other metabolic pathways, including the breakdown of the branched chain amino acids isoleucine and valine (Figure 7A-Supplementary file 3). Recent metabolomics data in patients with mutations in ECHS1 (the homolog of *C. elegans ech-6*) and HIBCH (the homolog of *C. elegans hach-1*) revealed elevated levels of acrylyl-CoA, a propionate shunt intermediate, in addition to the expected valine breakdown intermediates (Peters et al., 2014; 2015) (Figure 7A). Further, patients with mutations in ALDH6A1 (the homolog of *C. elegans alh-8*) exhibit elevated levels of 3-HP as well as elevated levels of valine breakdown intermediates (Marcadier et al., 2013). Taken together, these observations suggest that the closest human homologs of several *C. elegans* propionate shunt genes may have conserved roles in propionate breakdown in humans in addition to their known roles in other pathways.

We found that the closest human homologs of all five propionate shunt genes, ACADSB, ECHS1, HIBCH, ADHFE1 and ALDH6A1 are significantly co-expressed in both mouse and human across a large compendium of transcriptomic data in the SEEK database (Figure 7B) (Wang et al., 2015). This suggests that, like their *C. elegans* counterparts, these human genes may be co-regulated at the transcriptional level. Remarkably, the expression of ADHFE1, HIBCH, and to a lesser extent ECHS1 and ALDH6A1, is upregulated in response to propionate in HepG2 cells (Figure 7C). These data suggest that the regulated response of these genes to propionate at the gene expression level is, at least to some extent, conserved between *C. elegans* and humans.

## Discussion

In this study, we use a combination of synthetic genetics, network analysis, metabolomics and ^13^C carbon tracing to identify a propionate breakdown shunt in *C. elegans.* We show that the shunt is transcriptionally activated when propionate accumulates under low dietary vitamin B12 conditions, or when the canonical propionate breakdown pathway is genetically perturbed.

### Vitamin-controlled metabolic network rewiring

To our knowledge, our study presents the first example of transcriptional metabolic network rewiring in which the catabolic route of a cellular metabolite is dictated by the presence or absence of a dietary vitamin, in this case vitamin B12. Other vitamins that have known roles in regulating gene expression include vitamins A and D, and this regulation is important for development, growth and homeostasis. However, vitamins A and D do not function directly in the metabolic network as cofactors of metabolic enzymes, and instead function more like hormones; in fact vitamin D can be synthesized endogenously. Vitamin D, via the vitamin D receptor (VDR), regulates mineral uptake (Carlberg and Seuter, 2009; Haussler et al., 2008), while vitamin A, via the retinoic acid receptor (RAR), regulates developmental programs as well as the enzymes that interconvert the regulatory version of retinoic acid and the trans-retinal version required by rhodopsin for light-sensing (D'Aniello and Waxman, 2015; di Masi et al., 2015).

Less is known about potential regulatory roles of other vitamins, including those that function as true enzyme cofactors in the metabolic network. However, gene expression profiling in mammalian cells has revealed transcript-level responses to vitamins B1 (thiamine) (Fraser et al., 2012; Liu et al., 2004) (Tanaka et al., 2007), B2 (riboflavin) (Nakano et al., 2011), B3 (nicotinamide/niacin) (Choi et al., 2011; Couturier et al., 2014; Giammona et al., 2006), B6 (pyridoxal 5′ phosphate, PLP) (Toya et al., 2012; Zhang et al., 2014), B9 (folic acid) (Barua et al., 2014; Champier et al., 2012; Lin et al., 2011), C (ascorbic acid) (Canali et al., 2014; Jun et al., 2011; Takahashi et al., 2014), and E (tocopherol/tocotrienols) (Landrier et al., 2010; Makpol et al., 2013; Mustacich et al., 2009). The mechanisms behind, and consequences of, these observed vitamin-induced gene expression changes have yet to be elucidated. Our study indicates that, in *C. elegans*, low vitamin B12 leads to accumulation of the short chain fatty acid propionate due to reduced flux through the B12-dependent propionate breakdown pathway. It remains to be determined whether vitamin B12 is directly sensed similar to vitamins A and D, or whether propionate or perhaps one of its derivatives is the sole proxy regulator (see below).

In mammals, propionate and other short chain fatty acids produced by the gut microbiota provide numerous benefits to the host, not only as nutrient sources that fuel colonocytes, but also potentially to inhibit cancer cell proliferation, induce cancer cell apoptosis (Emenaker et al., 2001; Hinnebusch et al., 2002), and reduce inflammation (Louis et al., 2014). However, excess propionate accumulation, which occurs in patients with propionic- or methylmalonic acidemia, is toxic. It is possible that the metabolic network rewiring that we observe in *C. elegans* in response to the vitamin B12/propionate axis has evolved not only to optimize energy yield from propionate depending on the presence or absence of B12, but also to prevent toxic propionate buildup. This represents a novel example of built-in metabolic network flexibility to mitigate the toxic accumulation of an endogenous metabolite.

### The biological function of metabolic network rewiring by vitamin B12

What is the biological function of metabolic network rewiring by the vitamin B12/propionate axis? A loss of propionate breakdown capability, in double Δ*pcca-1*/Δ*acdh-1* mutants, or in Δ*acdh-1* mutants on a very low vitamin B12 diet (*E. coli* OP50 grown on soy peptone) is not compatible with viability (this study). Vitamin B12 is exclusively produced by bacteria, and studies of microbial communities have found that only 20–30% of community members synthesize vitamin B12 (Sañudo-Wilhelmy et al., 2014). *C. elegans* is found all over the world in temperate climates and is likely to feed on a variety of bacterial species (Félix and Duveau, 2012). Our data suggest that the ability to catabolize propionate whether or not vitamin B12 is provided by the diet may provide the animal with the metabolic flexibility to survive in different dietary conditions, thus providing a selective advantage and evolutionary benefit.

On vitamin B12-replete bacterial diets, such as *Comamonas aquatica*, expression of the propionate shunt is greatly reduced, indicating that the canonical vitamin B12-dependent propionate breakdown pathway is preferred. We speculate that this may be because the canonical pathway is more efficient at metabolizing propionyl-CoA than the propionate shunt due to the high redox potential between propionyl-CoA and acrylyl-CoA, the first shunt pathway intermediate (Sato et al., 1999). Other advantages of the canonical pathway over the shunt include the use of fewer enzymes, and the lack of production of highly toxic intermediates (e.g., acrylyl-CoA).

While all of the five genes identified in this study as propionate shunt members lead to similar phenotypes when mutated or knocked down (sensitivity to propionate-induced toxicity and at least partial lethality on B12-deficient diets), the severity of these phenotypes differs depending on the gene disruption. This could potentially be explained by different levels of reactivity (and therefore toxicity) among the intermediates in the pathway, which may accumulate to different levels depending on which enzyme is disrupted. For instance, *ech-6* knockdown results in a very severe phenotype, likely due to the accumulation of its substrate acrylyl-CoA, which is highly toxic. Simultaneously, the buildup of substrates containing CoA could lead to widespread metabolic impairment due to CoA sequestration (Mitchell et al., 2008). Additionally, several enzymes that function in the shunt may also have roles in isoleucine and valine breakdown, and may therefore be pleiotropic. It should be mentioned that the Δ*hphd-1* and Δ*alh-8* deletion mutants used in this study may not be null since they are both partial locus deletions, and this may explain the less severe phenotypes observed for these mutants compared with the null Δ*acdh-1* mutant. It is also possible that *C. elegans* can (somewhat) tolerate B12 deficiency with only a partially intact shunt consisting of the first three reactions, or that there are other unidentified (partially) redundant enzymes that can compensate for loss of *hphd-1* and *alh-8*.

### Transcriptional rewiring of *C. elegans* metabolism

How does *C. elegans* rewire its metabolic network in response to the vitamin B12/propionate axis? Each of the five genes that encode enzymes of the propionate shunt is repressed by vitamin B12 and activated by propionate. By using a GFP reporter driven by 1.5 kb of *acdh-1* promoter DNA we previously found that GFP levels are high when animals are fed bacterial diets low in vitamin B12, whereas GFP is greatly reduced when the animals are fed bacteria that synthesize high levels of vitamin B12, or upon direct supplementation of vitamin B12 (MacNeil et al., 2013; Watson et al., 2013; Watson et al., 2014). This demonstrated that the response of *acdh-1* occurs at the level of transcriptional regulation. Vitamin B12 is not sufficient to repress the *acdh-1* promoter when enzymes within the B12-dependent propionate breakdown pathway are genetically perturbed, or when excess propionate is added to the media (Watson et al., 2014). Therefore, we propose that the *C. elegans* gene regulatory network activates *acdh-1* expression in response to the buildup of propionate, which occurs when this vitamin is in low supply.

We have previously identified more than 50 *C. elegans* transcription factors that regulate *acdh-1* (MacNeil et al., 2015; Watson et al., 2013), including the nuclear hormone receptor NHR-10 that directly binds its promoter (Arda et al., 2010). Future studies will reveal which of these transcription factors mediate the response to propionate and/or vitamin B12. Nuclear hormone receptors utilize binding to small molecule ligands to regulate gene expression. For instance, VDR directly interacts with vitamin D, and RAR binds biologically active forms of vitamin A (Carlberg, 1999). While humans have 48 nuclear hormone receptors, *C. elegans* has more than 270 (Reece-Hoyes et al., 2005). It is tempting to speculate that one or more *C. elegans* nuclear hormone receptors directly respond to propionate, or its CoA derivative propionyl-CoA.

### Similarities and differences between propionate breakdown in *C. elegans* and humans

Several lines of evidence indicate that humans also utilize a propionate detox shunt, at least to some extent. First, the detection of the unique shunt intermediate 3-HP is used as a diagnostic marker for propionic- and methylmalonic acidemia in newborns. Since 3-HP is not predicted as an intermediate in any other metazoan pathway, this finding suggests that a propionate shunt may also be functional in humans. Second, while the human homologs of several *C. elegans* shunt enzymes have well-established functions in the breakdown of branched chain amino acids, their genetic perturbation also results in the accumulation of propionate shunt intermediates. For instance, recent metabolomic analyses of patients with mutations *ECHS1* and *HIBCH* revealed not only elevated upstream intermediates from valine catabolism, but also acrylyl-CoA, a unique intermediate from propionate oxidation (Peters et al., 2014). Interestingly, global metabolomics has identified 3-HP in healthy individuals (Bouatra et al., 2013; Guneral and Bachmann, 1994). This finding suggests that the propionate shunt may also be active to some degree when the canonical pathway is functional and thus may be part of central metabolism in humans.

It is important to note that, in spite of evidence supporting alternative propionate breakdown mechanisms in humans, patients with an impaired canonical propionate breakdown pathway are very sick and must strictly adhere to diets low in the amino acids that are broken down to propionyl-CoA. This indicates that, in humans, alternative propionate catabolism routes are not sufficient to maintain propionate levels below the toxic threshold.

### Identification of HPHD-1 as enzyme EC1.1.1.59

Perhaps the most interesting gene we identified as a participant in the propionate shunt is *hphd-1*, which is the one-to-one ortholog of human ADHFE1. *hphd-1* is the first metazoan gene to be associated with the reaction catalyzed by 3-hydroypropionate dehydrogenase (EC 1.1.1.59), which converts 3-HP to MSA. ADHFE1 is thought to metabolize a structural analog of 3-HP, GHB, which is commonly known as a recreational drug. However ADHFE1 is not assigned by KEGG or BRENDA enzyme databases to any endogenous metabolic pathway. ADHFE1 is unique in that, unlike most dehydrogenases that transfer electrons from their substrates to NAD or FAD, it transfers electrons to the TCA cycle intermediate α-ketoglutarate, thereby producing (D)-2-hydroxyglutarate (Struys et al., 2005), a putative oncometabolite (Dang et al., 2009; Kranendijk et al., 2010). Interestingly, other than neomorphic isocitrate dehydrogenase (IDH) enzyme mutants found in many cancers, ADHFE1 is the only known enzyme to naturally produce (D)-2-hydroxyglutarate (Struys et al., 2005). Currently, no patients have been identified with mutations in ADHFE1 so there is no metabolomics data available to determine which metabolites build up in humans lacking ADHFE1 enzyme function. However, our *C. elegans* mass spectrometry data in mutants lacking *hphd-1* revealed greatly elevated 3-HP levels (Figure 5), and, since *hphd-1* and ADHFE1 are clear one-to-one orthologs, ADHFE1 is a good candidate to function in propionate oxidation in humans directly downstream of 3-HP.

## Materials and methods

### *C. elegans* strains

N2 (Bristol) was used as the wild type strain, and animals were maintained as described (Brenner, 1974). *pcca-1(ok2282), acdh-1(ok1489), mce-1(ok243)* and *hphd-1(ok3580)* strains were provided by the *C. elegans* Gene Knockout Consortium and were backcrossed 3 times against N2 prior to assays. The *hphd-1*(ok3580) allele removes only part of the C-terminus of the protein and may not be a complete loss-of-function mutation. For a diagram of deletion mutant loci, see Figure 4B and for a full list of genotyping primers refer to Supplementary file 4.

### Propionate toxicity assays

Approximately 100 synchronized L1s (hatched overnight, 20 hr post-bleach) were added to *E. coli* OP50-seeded 35 mM NGM (bactopeptone) agar plates containing various concentrations of pH-neutralized propionic acid. Each dose tested included four technical replicates. After 72 hr, un-arrested survivors (animals that had developed past L1 stage) were counted. Dose response curves were fit to the raw data using the following equation:

$$Y=Bottom+(Top-Bottom)/(1+10^{\wedge }((LogLD50-X{)}^{*}HillSlope))$$

The dose required to kill 50% of the population (LD50) was calculated according to the fitted dose response curves. Toxicity assays were performed in biological triplicate, and the average LD50’s are plotted +/- SEM. To obtain enough viable Δ*acdh-1* mutant animals for these assays, 64nM B12 was supplemented to animals two generations prior to assay.

In the larval lethality quantifications, animals were fed for one generation on *E. coli* OP50 supplemented with 64nM B12, and then grown for one generation on *E. coli* OP50, *E. coli* OP50 +B12 or *Comamonas aq.* DA1877. Offspring were harvested and live and dead L1s and embryos were quantified following a 24 hr arrest.

### RNAi screen

A list of metabolic enzyme domain-containing genes was manually curated based on KEGG and WormBase databases, and available metabolic gene-targeting clones in the ORFeome RNAi library were re-arrayed in 96 well format. See Supplementary file 2 for the gene list. RNAi experiments were performed as follows: 24-well NGM (bactopeptone) agar plates containing 1 mM IPTG and 1 mM Ampicillin were seeded with one dsRNA-expressing *E. coli* HT115 clone per well the night before use. A separate set of plates containing 30 mM pH-neutralized propionate was also prepared and seeded with the same HT115 clones. The HT115 cultures were prepared by seeding 1 mL fresh LB + Ampicillin with 50 μL overnight culture, growing at 37^o^C for 6 hr, then centrifuged and resuspended in 150 μL LB + Ampicillin. 30 μL of this resuspended culture was placed in the center of NGM wells in the 24-well plates. Wild type and Δ*pcca-1* mutants were cultivated on *E. coli* OP50, and eggs were harvested by bleaching, and hatched overnight in M9 media (20 hr), and synchronized L1s were added to prepared plates. Animals were observed after three days to observe effects in the 1^st^ generation, and after six days to observe lethality in the 2^nd^ generation.

### *C. elegans* qRT-PCR experiments

Animals were synchronized by L1 arrest and grown on plates containing bactopeptone and various doses of B12 and/or propionate, seeded with *E. coli* OP50. Approximately 1500 adult animals were harvested for each condition, in biological duplicate. Animals were washed in M9 buffer, and total RNA was isolated using Trizol (Invitrogen) followed by DNAseI treatment and cleanup using Qiagen RNeasy columns. cDNA was prepared from RNA using oligo-dT and Mu-MLV enzyme (NEB). Primer sequences for quantitative RT-PCR (qRT-PCR) were generated using the GETprime database (Gubelmann et al., 2011) and are listed in Supplementary file 4. qPCR was performed in technical triplicate per gene per condition using the Applied Biosystems StepOnePlus Real-Time PCR system and Fast Sybr Green Master Mix (Invitrogen). Relative transcript abundance was determined using the ΔΔCt method (Schmittgen and Livak, 2008), and normalized to averaged *ama-1* and *act-1* mRNA expression levels.

### *C. elegans* liquid culture

Synchronized animals were cultivated on 15 cm NGM plates seeded with *E. coli* OP50, and bleached after 3 days. Bleached eggs were washed three times in M9, then allowed to hatch for 20 hr. 1 million synchronized L1s were added to 400 mL liquid NGM in a 2L Erlenmeyer flask, containing concentrated *E. coli* OP50 bacteria from 500 mL overnight LB culture, and total volume was adjusted to 450 mL with M9. Some flasks contained 20 mM pH-neutralized propionic acid. Flasks were kept at 20°C, shaking gently at 100 rpm. Each day concentrated bacteria were added to the flasks to feed the worms. Adult animals were collected (after 3 days of development for N2, and four days for the mutants) and washed 2 times in M9 in sterile Imhoff settling cones. The final pellet was flash-frozen and stored at −80°C until extraction.

### *C. elegans* metabolite extraction

Cell extracts were obtained by re-suspending the frozen *C. elegans* or bacterial pellets in 4 mL of 5% trichloroacetic acid (TCA). Cell suspensions were homogenized in a Polytron PT 1300 for 2 min at 20,000 rpm and neutralized with 1 mL of 2M of potassium monoacid phosphate. The samples were centrifuged at high speed for 10 min at 4°C and immediately injected for propionyl-CoA determination. The pellets were stored for protein quantification using the bicinchoninic acid method (Thermo Scientific Pierce Protein BCA Kit). For 3-HP measurements, the cell extract was desiccated in a Speedvac and the resulting pellet was resuspended in the same volume of methanol. Then, the samples were centrifuged at high speed for 10 min at 4°C. For *E.coli* metabolite extraction, the cell extracts were obtained as mentioned above except that bacteria cells were disrupted by sonication for 2 min using intervals of 15 s of sonication followed by 15 s for cooling.

### LC-MS/MS quantification

The quantification of metabolites was performed using a LC-MS/MS system consisting of an ultra-high pressure LC system (Agilent 1290) online coupled to a Triple Quadrupole mass spectrometer equipped with an electrospray ionization source (Agilent 6460). Propionyl-CoA was separated using a column Zorbax Eclipse Plus C18 Rapid Resolution HD 2.1 × 50 mm 1.8 Micron (Agilent) at 30°C. The mobile phase was composed of Buffer A: 10 mM tributylamine, 15 mM acetic acid and 5% methanol; and Buffer B: 100% methanol. The flow rate was 0.5 mL/min and the gradient method consisted of: 0–0.25 min, 2.5% B; 0.25–0.5 min, 2.5–30% B; 0.5–5 min, 30–70%B; 5–5.25 min, 70–100% B; 5.25–6.25 min, 100% B; 6.25–7 min, 2.5% B; 7–8 min 2.5% B. The 3-hydroxypropanoic acid was separated using a column Zorbax Eclipse Plus C18 Rapid Resolution HD 2.1 × 100 mm 1.8 Micron (Agilent) at 30 C. The mobile phase was composed of Buffer A: 0.1% formic acid in water; and Buffer B: 100% methanol. The flow rate was 0.35 mL/min and the gradient consisted of: 0–3 min, 5% B; 3–4 min, 5–70% B; 4–5.25 min, 70%B; 5.25–5.5 min, 70–100% B; 5.5–6.5 min, 100% B; 6.5–7 min, 5% B; 7–7.5 min 5% B. Q1/Q3 (MRM) transitions, ion source and collision energy settings were optimized according to the metabolites and were: 91->73, 25 eV; 824.2->317.1, 25 eV; and 92->74, 25 eV (in positive mode), for 3-HP, propionyl-CoA and ^13^C labeled 3-HP, respectively. Ion source settings were as follows: gas temperature, 300 C: gas flow, 8L/min; nebulizer 50 psi (Nitrogen); sheath gas temperature, 200 C: sheath gas flow, 11 L/min, capillary 3500 V and nozzle voltage, 500 V. We confirmed the peak identity of 3-HP by matching retention time, mass/charge ratio and MS/MS fragmentation spectra to a chemically synthesized 3-HP standard.

### CRISPR/Cas9 genome editing

The *alh-8* mutant was generated by dual sgRNA directed-deletion (Chen et al., 2014). We used a co-CRISPR strategy, which includes *unc-22* as a CRISPR marker to enhance detection of genome-editing events (Kim et al., 2014). The target sequences were manually derived to conform to the sequence N19NGG near the 5’ end of *alh-8*. Two target sequences were chosen: CCGCCCATCTCTTGTGATTTTC and CTGTGCGACAGTTGTCGTATGG. We designed forward and reverse oligos containing the N19 sequence and ends of *Bsa*I recognition sites. The forward and reverse oligos were annealed and ligated to *Bsa*I-digested pRB1017 vector (Arribere et al., 2014). The *alh-8* sgRNA plasmids were prepared using a PureLink Quick Plasmid Miniprep Kit (Invitrogen). The other co-injected DNA vectors were purified using a Qiagen midiprep kit. The DNA mixture used in microinjection contained *Peft-3::Cas9* vector, *pRF4::rol-6(su1006), unc-22* sgRNA vector (all gifts from the Mello lab) and two *alh-8* sgRNA vectors, each with a concentration of 40 ng/µl. Approximately 20 young adult hermaphrodite worms were injected. After recovering from injection, each worm was placed onto an individual *E. coli* OP50 plate. After 2–3 days, the F1 rollers (dominant phenotype indicating presence of the *pRF4::rol-6(su1006)* construct) were picked onto new plates. F1s with twitcher F2s were genotyped by PCR for mutations in *alh-8*. The PCR primers are outside of the sgRNA-targeted region. Forward primer: TTCAATGTTCGCGTGTATTTTG; Reverse primer: TCAGCGAGCTTCTTCATGT. The amplicons with smaller size than wild type amplicons were reconfirmed by sequencing. Forward primer: ATTCGAAACGTGATCAGTAATG; Reverse primer: CTCTCTTGATCAAGGCTTGA. A mutant animal with a ~400 bp deletion (23 bp indel and 399 bp deletion) was chosen for further analysis, and was outcrossed with N2 three times before use in phenotypic assays.

### Network analysis - WISP

The WISP tissue-specific functional networks were built using a semi-supervised regularized Bayesian approach that integrated 56,179 expression- and interaction-based measurements across 174 genome-level datasets [http://wisp.princeton.edu; Yao et al., in preparation, V. Yao, personal communication, June 2016]. Using the intestine network, we found genes that were tightly connected to *acdh-1, ech-6* and *hach-1* even after adjusting for average network connectivity, which improved the specificity of the retrieved genes to our seed genes.

### Network analysis – SEEK

The SEEK (seek.princeton.edu) and modSEEK search engines have compiled thousands of publicly available expression datasets and given a gene set query, weights datasets by relevance (using a cross-validation method) and calculates a weighted coexpression score for every other gene in the genome to the gene set according to the dataset relevance weights. For every gene set, we can thus use the leave-one-out approach to calculate average weighted coexpression scores for the entire set. To construct null distributions for these 5-gene queries, we compared with average weighted coexpression scores based on random sets (n = 10,000) from (1) all genes with sufficient data; (2) all genes that show enzymatic activity (as indicated by known annotation to the catalytic activity GO term); (3) all genes in similar gene families / related pathways (members of *acdh, ech, alh* families, as well as those known to participate in branched chain amino acid breakdown, the canonical propionyl-CoA breakdown pathway, and the TCA cycle).

### Tissue culture

HepG2 cells were seeded in 6 well plates at 0.6 × 10^6^ cells/ml in 3 ml DMEM plus 1% FBS with or without 50 mM propionic acid. Cells were incubated for 48 hr at 37°C, 5% CO_2_ and 65% relative humidity. Cells were washed two times in PBS and before proceeding to Trizol lysis for RNA extraction. qRT-PCR was performed as described above, using actin and GAPDH to normalize expression levels.

## Additional files

Supplementary file 1. Summary of *acdh-1* genotypes in F1 generation of P0 *pcca-1 -/-; acdh-1* +/- animals. The *acdh-1* genotype (homozygous, heterozygous, or wild type) as determined by PCR for each F1 animal chosen from P0 *pcca-1* +/+; *acdh-1* +/- or *pcca-1 -/-; acdh-1* +/- parents is shown, as well as an indication of whether the F1 animal laid non-viable embryos among the F2 offspring. **DOI:** http://dx.doi.org/10.7554/eLife.17670.011

Supplementary file 2. Metabolic gene lists. A manually curated list of putative metabolic genes in *C. elegans*. The 836 genes that were screened as part of the metabolic RNAi library are indicated. **DOI:** http://dx.doi.org/10.7554/eLife.17670.012

Supplementary file 3. Evidence summary for dual roles of human orthologs in propionate oxidation shunt and other metabolic pathways. **DOI:** http://dx.doi.org/10.7554/eLife.17670.013

Supplementary file 4. Genotyping and qPCR primers. **DOI:** http://dx.doi.org/10.7554/eLife.17670.014
